# Thromboembolic and atherosclerotic cardiovascular events in inflammatory bowel disease: epidemiology, pathogenesis and clinical management

**DOI:** 10.1177/17562848211032126

**Published:** 2021-07-28

**Authors:** Jasmijn A. M. Sleutjes, Jeanine E. Roeters van Lennep, C. Janneke van der Woude, Annemarie C. de Vries

**Affiliations:** Department of Gastroenterology and Hepatology, Erasmus Medical Center, Rotterdam, the Netherlands; Department of Internal Medicine, Erasmus Medical Center, Rotterdam, the Netherlands; Department of Gastroenterology and Hepatology, Erasmus Medical Center, Rotterdam, the Netherlands; Department of Gastroenterology and Hepatology, Erasmus Medical Center, Dr. Molewaterplein 40, Room Na-618, Rotterdam 3015GD, The Netherlands

**Keywords:** cardiovascular disease, drugs, genetics, inflammation, inflammatory bowel disease, lipids, morbidity, mortality, traditional risk factors

## Abstract

Inflammatory bowel disease (IBD) is associated with an increased risk of cardiovascular disease (CVD). The increased risk of CVD concerns an increased risk of venous thromboembolism (VTE), atherosclerotic cardiovascular disease (ASCVD) and heart failure (HF), at corresponding relative risks of 2.5, 1.2 and 2.0, respectively, as compared with the general population. Especially young patients under the age of 40 years run a relatively high risk of these complications when compared with the general population. Chronic systemic inflammation causes a hypercoagulable state leading to the prothrombotic tendency characteristic of VTE, and accelerates all stages involved during atherogenesis in ASCVD. Increased awareness of VTE risk is warranted in patients with extensive colonic disease in both ulcerative colitis and Crohn’s disease, as well as during hospitalization, especially when patients are scheduled for surgery. Similarly, critical periods for ASCVD events are the 3 months prior to and 3 months after an IBD-related hospital admission. The increased ASCVD risk is not fully explained by an increased prevalence of traditional risk factors and includes pro-atherogenc lipid profiles with high levels of small dense low-density lipoprotein cholesterol particles and dysfunctional high-density lipoprotein cholesterol. Risk factors associated with HF are location and extent of inflammation, female sex, and age exceeding 40 years. A dose-dependent increase of overall CVD risk has been reported for corticosteroids. Immunomodulating maintenance therapy might reduce CVD risk in IBD, not only by a direct reduction of chronic systemic inflammation but possibly also by a direct effect of IBD medication on platelet aggregation, endothelial function and lipid and glucose metabolism. More data are needed to define these effects accurately. Despite accumulating evidence on the increased CVD risk in IBD, congruent recommendations to develop preventive strategies are lacking. This literature review provides an overview of current knowledge and identifies gaps in evidence regarding CVD risk in IBD, by discussing epidemiology, pathogenesis, and clinical management.

## Introduction

Cardiovascular disease (CVD) is a major cause of morbidity and mortality worldwide, despite significant advances in cardiovascular risk management over the past decennia.^1^ CVD can be divided into venous and arterial disease, which both have a distinct pathogenic pathway, epidemiology, risk profile, diagnostics, and therapeutic strategy. Inflammatory bowel disease (IBD) is a chronic inflammatory condition affecting the gastrointestinal tract and comprises ulcerative colitis (UC) and Crohn’s disease (CD). Similar to other chronic inflammatory diseases, including rheumatoid arthritis, systemic lupus erythematosus, and psoriatic arthritis, IBD is associated with an increased risk of venous thromboembolism (VTE) and atherosclerotic cardiovascular disease (ASCVD;^2–5^ Table 1). The overall underlying explanation for the association between IBD and CVD seems to be linked to the immunological processes in both chronic systemic inflammation and atherogenesis. The aim of this paper was to review current knowledge on epidemiology, pathogenesis, diagnosis, and treatment of both VTE and ASCVD in IBD patients.

**Table 1.** Relative risk of venous and arterial CVD in different chronic inflammatory diseases as compared with the general population following meta-analyses.

	VTE	HF	IHD	CeVD	PAD	Mortality
RA	1.94 (1.79–2.10)*	1.87 (1.47–2.39)*	1.26 (1.04–1.52)	1.90 (1.70–2.10)	NS	1.30 (1.20–1.40)
SLE	3.55 (2.69–4.69)*	1.27 (0.65–2.49)*	3.39 (2.15–5.35)	1.95 (1.52–2.53)	4.10 (1.5–11.6)**	2.72 (1.83–4.04)
PsA	1.20 (1.03–1.40)*	1.50 (1.10–2.00)	1.68 (1.31–2.15)	1.22 (1.05–1.41)	NA	1.62 (1.31–2.12)
IBD	2.20 (1.83–2.65)	2.03 (1.35–3.03)	1.17 (1.07–1.27)	1.25 (1.08–1.44)	NS	NS

* Studies reporting hazard ratios instead of RR; **studies reporting odds ratio instead of RR. The parenthetical numerals are the 95%CI.

95%CI, 95% confidence interval; CD, Crohn’s disease; CeVD, cerebrovascular disease; HF, heart failure; IBD, inflammatory bowel disease; IHD, ischemic heart disease; NA, not available; NS, non-significant; PAD, peripheral artery disease; PsA, psoriatic arthritis; RA, rheumatoid arthritis; RR, relative risk; SLE, systemic lupus erythematosus; UC, ulcerative colitis; VTE, venous thromboembolism.

## Venous thromboembolism

### Epidemiology

The risk of VTE is 2.5-fold higher in the IBD population as compared with controls, as has been shown in two meta-analyses of case-control studies, prospective cohort studies and population-based cohort studies.^6,7^

Up to 90% of VTE cases concern deep venous thrombosis [DVT; relative risk (RR) 2.42; 95% confidence interval (95%CI) 1.78–3.30] or pulmonary embolism (PE; RR 2.53; 95%CI 1.95–3.28). In the remaining 10% of VTE cases, other locations are affected, such as the mesenteric and cerebral veins.^6–8^ The risk of recurrent VTE within 5 years after discontinuation of coagulation therapy is higher among IBD patients as compared with controls (33.4% *versus* 21.7%, respectively; *p* = 0.010).^9^ Noteworthy, half of IBD patients had active disease at time of recurrent VTE. These data indicate that thromboprophylaxis may be considered during flares in IBD patients with a history of VTE.

### Risk factors

#### Patient characteristics

The incidence rates of VTE increase with age and absolute risk is higher in patients aged ⩾60 years as compared with IBD patients aged <60 years (54.6/10.000 person-years *versus* 8.9/10.000 person-years, respectively). Nevertheless, relative risks in IBD patients as compared with controls are especially high in younger age groups, since IBD patients experience VTE at an earlier age as compared with controls.^10,11^ Among IBD patients below the age of 40 years the incidence rate ratio (IRR) is 6.02 (95%CI 3.92–9.12) as compared with age- and sex-matched controls.^10^ Also, in pediatric and adolescent populations the relative risk of VTE is more than two to threefold higher [hazard ratio (HR) 6.6; 95%CI 3.3–13.2 in patients aged ⩽20 years *versus* HR ranging from 1.6 to 2.8 in older age groups].^12^ No evident association between VTE risk and sex has been observed. In obese IBD patients, an increased prevalence of DVT and PE has been observed as compared with non-obese patients, according to preliminary data.^13^

#### Disease characteristics

The extent and location of IBD are associated with VTE risk. According to a retrospective study of 60 patients with confirmed IBD and DVT, 76% of VTE cases occurred in UC patients with pancolitis, and 24% of VTE cases occurred in patients with colitis limited to the left-sided colon or proctitis. In 40 CD patients, 79% of VTE cases occurred in patients with ileocolonic or colonic involvement, and 21% in patients with ileal disease.^14^

VTE risk is twice as high as compared with matched controls in quiescent IBD (HR 2.1; 95%CI 1.6–2.9; 1.4/1000 person-years), and increases to 8.5-fold in patients with active IBD (HR 8.4; 95%CI 5.5–12.8; 9.0/1000 person-years).^15^ The majority of VTEs during disease flares occur in outpatients in both absolute and relative numbers (non-hospitalized periods: HR 15.8; 95%CI 9.8–25.5; 6.4/1000 person-years *versus* during hospitalization: HR 3.2; 95%CI; 37.5/1000 person-years, respectively).^15^ VTE risk of hospitalized IBD patients is increased regardless of the indication for hospital admission, e.g. IBD flare, surgery or non-IBD-related reasons, probably related to in-hospital immobilization.^16^ Among hospitalized CD patients, intra-abdominal fistulizing disease was independently associated with VTE [odds ratio (OR) 1.39; 95%CI 1.13–1.70].^17^

#### Traditional VTE risk factors

Although VTE risk in IBD is increased independently of known clinical risk factors, i.e. long-distance journey, postoperative status, injury/immobility, pregnancy/delivery, oral contraceptives/hormone substitution, a cumulative effect of known clinical risk factors for VTE may be present.^18^

Data on VTE risk in IBD patients who use hormonal contraception specifically, are lacking. Based on an overall twofold increased VTE risk over baseline in women using estrogen-based methods, a cumulative effect of oral contraceptives and IBD may be relevant, especially in patients with active IBD. Therefore, individual counseling of patients on the optimal contraceptive method is recommended.^19^

Several studies indicate that intestinal resection increases the risk of both in-hospital and post-hospitalization VTE events when compared with non-IBD patients, resulting in rates of postoperative VTE between 0.6% and 8.9%. Surgery-related risk factors are emergency surgery, open procedure, longer operative time, ileostomy formation, anastomotic leak, and ileus.^20^ The indication for surgery, e.g. related to IBD or colorectal cancer, is not associated with VTE risk. Patient-specific risk factors for postoperative VTE are diagnosis of UC (higher risk as compared with CD), age above 65 years, and obesity.^20–22^

According to a meta-analysis of five cohort studies, risk of VTE during pregnancy and postpartum is two to three times higher in the IBD population as compared with controls (RR 2.13; 95%CI 1.66–2.73 and RR 2.61; 95%CI 1.84–3.69, respectively). In both periods, UC patients are more at risk than CD patients. During pregnancy, the risk of DVT is increased, whereas for PE, no significant increased risk has been demonstrated (RR 2.74; 95%CI 1.73–4.36 *versus* RR 1.81; 95%CI 0.81–4.05, respectively).^23^ Two studies addressed VTE in context of disease flares during pregnancy and showed that the pooled RR of pregnancy-associated VTE increased to 7.81 (95%CI 0.90–6.78).^24,25^

#### Genetic factors

IBD is not associated with hereditary coagulation disorders predisposing to thrombosis.^26,27^ The prevalence of mutations in genes encoding factor V Leiden, prothrombin G20210A, and methylenetetrahydrofolate reductase (MTHFR) is comparable in VTE patients with IBD and controls.^26–29^ A recent genome-wide association study (GWAS) and whole-exome sequencing study investigated and defined high genetic risk for VTE as the presence of multiple VTE genetic variants or at least one pathogenic variant.^30^ This high genetic risk was present in approximately one in seven IBD patients. VTE risk in IBD patients was significantly associated with both high polygenic risk scores and carriage of thrombophilia pathogenic variants (OR 3.13 and 2.11, respectively). An additional effect of genetic burden on VTE risk was suggested and associated with shorter time between IBD diagnosis, and VTE event and thrombosis at multiple sites. In general, analysis of genetic or acquired thrombophilia disorders may be disregarded after a diagnosis of VTE in patients with active IBD, unless a family history of VTE is present.^31^

### Pathogenesis

Ample evidence from basic investigations supports the hypothesis of a hypercoagulable state in IBD, including the observations of increased levels of procoagulants, vascular endothelial dysfunction, decrease in anticoagulants and alterations in the fibrinolytic system leading to a prothrombotic tendency.^11,32^ In addition, several studies have demonstrated an increased prevalence of antiphospholipid antibodies among IBD patients as compared with controls.^33,34^ Hypercoagulability has been observed both in quiescent and active IBD, and is substantiated by data on levels of the coagulation and fibrinolytic cascade.^35,36^ For instance, thrombocytes showed a higher status of activation leading to an increased tendency to intravascular aggregation regardless of disease activity.^37^ The hypercoagulable state is probably most pronounced during IBD disease flares, during which the procoagulation factors V, VII, VIII, X, XI, XII, von Willebrand factor, and fibrinogen are increased, and important drivers of anticoagulation, i.e. protein S and antithrombin, are decreased, and thrombocytosis and thrombocyte dysfunction have been observed.^38–40^

### IBD drugs and VTE risk

#### Aminosalicylates

Clinical studies designed to evaluate the effect of aminosalicylic acid (5-ASA) on VTE risk are not available. Platelets isolated from IBD patients receiving 5-ASA showed a reduction in both spontaneous and thrombin-induced platelet activation *in vitro*.^41^ No correlation was found between oral dosing and the level of platelet inhibition.

#### Corticosteroids

According to a systematic review and meta-analysis of observational studies, treatment with systemic corticosteroids during a flare is associated with a twofold increased risk of VTE as compared with patients not using corticosteroids (OR 2.20; 95%CI 1.70–2.86).^42^ Due to a substantial heterogeneity across selected studies, authors could not comment on confounding by hospitalization, immobilization or severity of the IBD flare. The association between corticosteroids and VTE risk may be dose dependent.^43^ This hypothesis is illustrated by the findings of a prospective study, in which IBD patients receiving high-dose steroids in tapering regimen showed a decrease in fibrin degradation products and stability in fragments of prothrombin.^44^ *In vivo* experimental studies in healthy individuals have shown that glucocorticoid treatment increases the levels of clotting factors and fibrinogen, but this finding has not been investigated in IBD patients.^45^

#### Immunomodulators: methotrexate and thiopurines

Clinical data directly addressing the effect of immunomodulators on the incidence of VTE are not available. Methotrexate elevates homocysteine levels by antagonization of folic acid.^46^ In IBD patients, the effect of methotrexate on homocysteine metabolism is counterweighted by use of folic acid.^47^ Thiopurines are hypothesized to lower VTE risk by the reduction of platelet aggregation *in vitro*, and subsequently, inhibition of platelet-leukocyte aggregation.^48,49^ Clinical data need to confirm this hypothesis.

#### Biologicals: TNFα antagonists, vedolizumab, ustekinumab

According to a meta-analysis of eight studies, anti-tumor necrosis factor alpha (TNFα) agents are associated with a significantly lower risk of developing VTE as compared with corticosteroids (OR 0.27; 95%CI 0.11–0.67).^42^ In a retrospective study, multivariate regression analysis of risk factors for VTE in IBD identified anti-TNFα therapy as a protective factor (OR 0.2; 95%CI 0.04–0.99).^50^ These findings may not only be explained by effective treatment of chronic inflammation, but may also be attributed to the hypothesis that TNFα accelerates thrombus formation *via* its direct association with endothelial dysfunction.^51,52^

Long-term *post hoc* analyses of the registration trials of vedolizumab as well as real-life cohort studies conclude an overall low VTE risk (UC < 1%, CD 1.5%).^53,54^ Pooled data from the phase II/III clinical trials showed no significant difference in VTE risk in CD patients treated with ustekinumab as compared with placebo (0.75/100 person years *versus* 0.34/100 person years, respectively).^55^ Only one real-life cohort study in 152 CD patients on ustekinumab reported one case of DVT after 1-year follow up.^56^

#### Janus kinase inhibitors: tofacitinib

After the publication of a safety warning for tofacitinib 10 mg twice daily in rheumatoid arthritis, VTE risk during tofacitinib treatment is under a magnifying glass.^57^ For UC, reported incidence rates of DVT and PE during tofacitinib treatment are not higher than expected: respectively, 0.04 patients/100 patient years and 0.16 patients/100 patient years.^58^ In addition, VTE cases have only been observed in UC patients on predominant dose of 10 mg twice daily, and all cases had additional risk factors for VTE alongside UC. While large post-marketing cohort data on safety are awaited, increased awareness among clinicians seems prudent, and alternative treatment strategies in patients at increased risk of VTE warrant consideration.

### Effect VTE drugs on inflammation in IBD

#### Anticoagulants

Since heparin possesses immunomodulatory and anti-inflammatory effects *via* TNFα inhibition, a number of small studies have evaluated the efficacy of both unfractionated heparin and low-molecular-weight heparins in UC.^59,60^ A meta-analysis of eight randomized controlled trials in 454 UC patients showed that subcutaneously administered heparin in UC is safe but has no additional benefit over conventional therapy with regard to efficacy (OR 0.78; 95%CI 0.50–1.21).^61^ In a double-blind, randomized trial, 141 UC patients taking extended colon-release heparin tablets achieved clinical and endoscopic improvement as compared with placebo.^62^ This finding remains unconfirmed. Therefore, there is no role for heparin as monotherapy or additional therapy in the management of UC.^63^ The effect of vitamin K agonist and direct oral anticoagulants on IBD inflammation are not addressed in available literature.

### Guidelines’ recommendations for VTE in IBD patients

The European Crohn and Colitis Organization (ECCO) guidelines describe recommendations on VTE management in IBD.^64^ In 2014, the Canadian Association of Gastroenterology (CAG) presented a consensus on the prevention and treatment of VTE in IBD.^16^ Other guidelines, including the American Society of Hematology (ASH) and European Society of Cardiology (ESC) regard IBD as a chronic, moderate risk factor for VTE.^65,66^

Guidelines recommend prompt diagnostic evaluation in the case of clinical suspicion of VTE. Thromboprophylaxis is indicated during hospitalization, and may be considered at discharge after hospitalization, after recent surgery and in outpatients with active IBD. The recommendation for thrombopropylaxis during hospitalization accounts for all IBD patients, regardless of the reason for admission. The duration of thromboprophylaxis is not specified for most clinical situations. In high-VTE-risk patients undergoing surgery without high risk of bleeding, CAG recommends extension of the pharmacologic prophylaxis during 4 weeks after discharge.

To date, no clinical trials address VTE treatment in IBD patients specifically. Both ECCO and CAG guidelines state that VTE treatment in IBD should follow the general antithrombotic therapy guidelines. Specific recommendations on the duration of anticoagulant therapy differ between guidelines. ASH recommends continuing antithrombotic therapy indefinitely after completion of primary treatment, unless comorbidities are present that predispose toward increased risk for bleeding complications (e.g. older age, history of bleeding, prior stroke, cancer, anemia, thrombocytopenia). In the CAG guideline, anticoagulant therapy is advised for a minimum of 3 months in the case of a first VTE. In case of a reversible provoking factor, recommendation is to only stop anticoagulant therapy 1 month after resolving the risk factor. Moderate to severe disease activity may be considered as a provoking factor for VTE. This implicates the indication of thrombopropylaxis in patients with a flare of IBD and a history of VTE, as mentioned previously.

## Atherosclerotic cardiovascular disease

### Epidemiology

The risk of ischemic heart disease (IHD) and cerebrovascular disease (CeVD) is significantly, though modestly, increased in IBD patients. A recent meta-analysis of 11 studies reported a pooled RR of 1.17 (95%CI 1.07–1.27), 1.12 (95%CI 1.05–1.21), and 1.25 (95%CI 1.08–1.44) for coronary heart disease (CHD), myocardial infarction (MI) and CeVD, respectively.^5^ IBD patients have twice the risk of heart failure (HF) as compared with the general population (HR 2.03; 95%CI 1.35–3.03).^67^ To date, an increased risk of peripheral artery disease has not been established.^68^ Cardiovascular mortality in IBD patients is not increased as compared with the general population (pooled standardized mortality ratios for CD: 1.01; 95%CI 0.90–1.14, and for UC: 0.93; 95%CI 0.86–1.01).^5^ Mortality is regarded as a more robust endpoint as compared with CVD events, being less sensitive to detection bias. Vice versa, mortality might underestimate the prevalence of ASCVD, since an increasing proportion of patients survive ASCVD due to significant advances in the prevention, diagnosis and treatment of ASCVD.

### Risk factors

#### Patient characteristics

Regarding risk factors for ASCVD in IBD, the relative risks of IHD and CeVD is most pronounced in female patients, and the risk of CeVD in patients below the age of 40 years.^5,68^ The observation of sex differences is probably related to differences in the prevalence and relative contribution of other risk factors. A potential explanation might be the greater contribution of chronic inflammation in the pathogenesis of ASCVD in women as compared with men. This is illustrated by the higher levels of C-reactive protein (CRP) in female patients with chronic inflammatory diseases.^69^ In line with this, CRP levels were positively correlated with the relative risk of CHD, independent of traditional cardiovascular risk factors.^70^ The contribution of sex hormones in development of ASCVD in IBD patients remains elusive. The increased relative risk of ASCVD in younger IBD patients might be the result from a younger age at IBD diagnosis accompanied by a more severe disease course, resulting in a prolonged exposure to chronic inflammation.^71^

#### Disease characteristics

Remarkably, the increased risk of IHD is already observed in the first year of IBD diagnosis (IRR 2.13; 95%CI 1.91–2.38), which may be explained by the commonly observed delay in IBD diagnosis.^72^ The degree of inflammation, both systemic (CRP levels) and clinical scores, was positively correlated to the development of IHD.^73^ Disease flares were associated with an increased risk of MI (RR 1.49; 95%CI 1.16–1.93) and stroke (RR 1.53; 95%CI 1.22–1.92).^71^ Critical periods for the development of ASCVD events are the 3 months prior to and 3 months after an IBD-related hospital admission.^74^ Furthermore, the extent and location of inflammation was associated with IHD risk, both in UC (pancolitis 6.32/1000 person years *versus* proctitis/left-sided 2.95/1000 person years) and CD (colonic disease 5.61/1000 person years *versus* ileum/ileocecal 4.35/1000 person years). As compared with controls, the subgroup of patients suffering from colonic involvement of IBD showed a threefold higher risk of developing MI.^67^

With regard to HF, a large Danish population study has shown that IBD patients with disease flares and persistent disease activity are 2.5 times more like to be admitted to the hospital due to HF as compared with IBD patients in disease remission.^75^ The risk of HF, caused by ischemic or dilated cardiomyopathy (77%) or diastolic dysfunction (23%), emerges more clearly in patients diagnosed with UC as compared with CD (adjusted HR 2.06; 95%CI 1.18–3.65 *versus* HR 1.73; 95%CI 0.98–3.07, respectively).^67^ Risk factors associated with HF were extent of inflammation (colonic disease in CD, pancolitis in UC), female sex, and age exceeding 40 years.^67^

#### Traditional ASCVD risk factors

**Figure 1.**
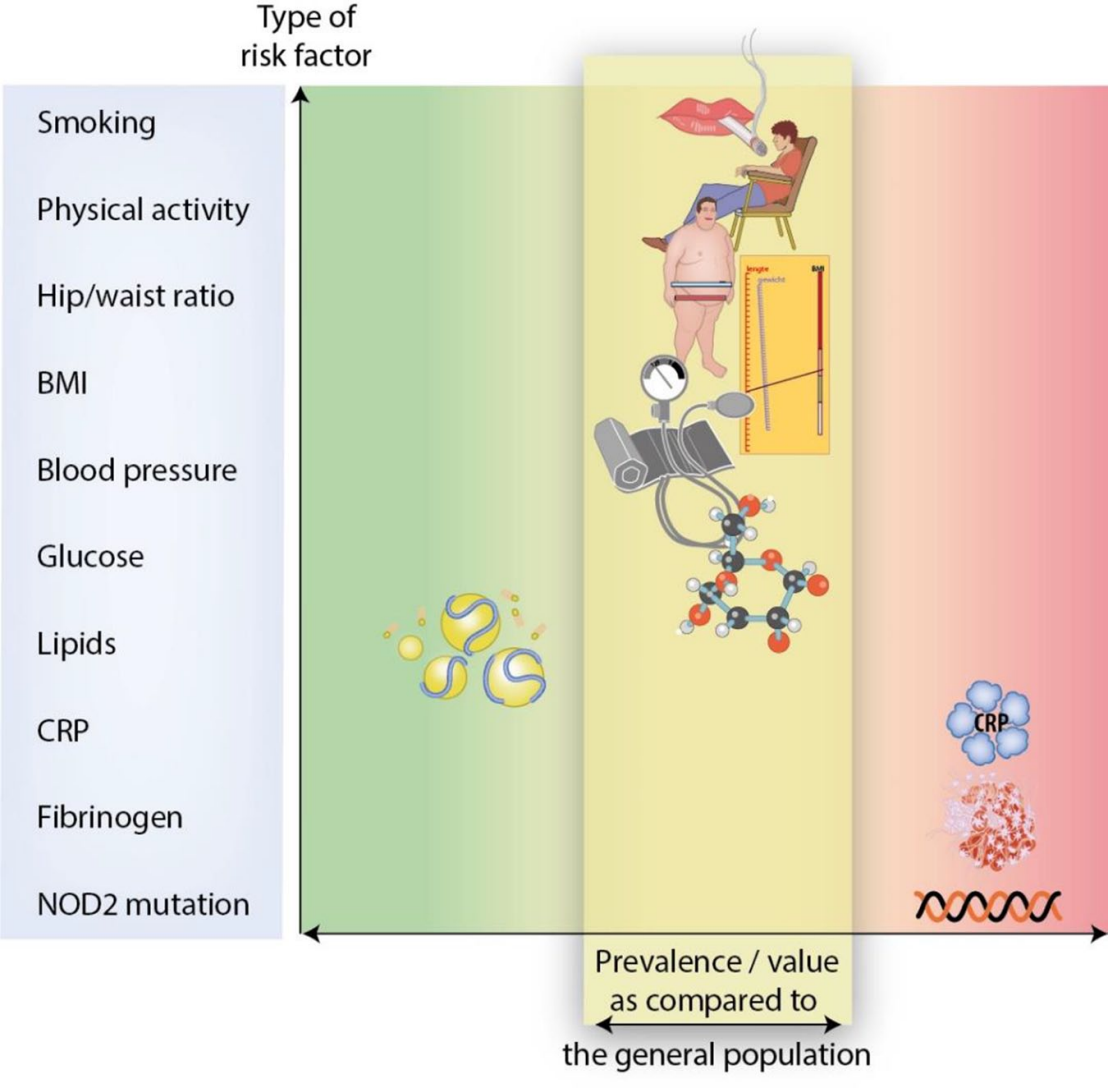
Prevalence of cardiovascular risk factors as compared with the general population. BMI, body mass index; CRP, C-reactive protein; NOD2, nucleotide-binding oligomerization domain-containing protein 2.

The increased ASCVD risk in IBD patients is not fully explained by an increased prevalence of traditional risk factors, according to available data. A large population study showed no increase in the traditional cardiovascular risk factors in IBD patients as compared with the general population. In this study, population blood pressure, total cholesterol (TC) and low-density lipoprotein cholesterol (LDL-c) levels were slightly lower in IBD patients. Other traditional risk factors including plasma glucose, body composition [body mass index (BMI) and hip and waist circumference] and active smoking were comparable in the IBD and general population (Figure 1). These findings were unaffected after excluding individuals taking lipid-lowering drugs.^76^ This observation has been confirmed by both cross-sectional and longitudinal cohort studies.^77,78^

The risk of diabetes mellitus (DM) type 2 is increased in IBD patients as compared with the general population [standardized incidence ratio (SIR) 1.54; 95%CI 1.49–1.60], independent of IBD diagnosis or sex.^79^ The prevalence of DM type 2 in IBD populations is increasing, illustrated by the finding that IBD patients diagnosed after 2014 have a higher risk of DM type 2 than patients receiving the diagnosis before the 21st century (SIR 1.79; 95%CI 1.67–1.91 *versus* 1.48; 95%CI 1.41–1.56, respectively, *p* < 0.001).^79^ A recent meta-analysis confirmed an elevated risk of DM in IBD, both UC and CD (OR 1.26; 95%CI 1.03–1.53).^80^ Insulin resistance and β-cell activity were shown to be significantly elevated in CD patients as compared with healthy controls.^81^

In general, IBD patients present with lower levels of TC and LDL-c, and no significant difference is seen with regard to high-density lipoprotein cholesterol (HDL-c) and triglyceride (TG) concentrations as compared with the general population.^76,82^ These lipid alterations were independent of disease activity. First, systemic inflammation, but also local inflammation of the intestine could play a role in the lower lipid levels during active disease. Second, chronic inflammation is associated with a more pro-atherogenic lipid profile characterized by small dense LDL-c particles and dysfunctional HDL-c losing its anti-inflammatory and cardioprotective properties.^82,83^ Moreover, intestinal surgery has an influence on the lipid metabolism. Greater length of resected ileum segment was inversely correlated with plasma TC and LDL-c levels.^84^ Evidence suggests that intestinal fat and cholesterol absorption is impaired in patients with an ileoanal anastomosis.^85^

Framingham Risk Scores (FRS), a model to predict the 10-year risk of developing ASCVD in the general population, are in conflict with the observed higher incidence and prevalence of ASCVD in IBD. For instance, the FRS were significantly lower in IBD patients with CHD as compared with non-IBD controls with CHD (8.1 plus −3.47 *versus* 10 plus −3.75, respectively, *p* = 0.001).^86^ This finding illustrates that traditional risk factors insufficiently account for the increased risk of ASCVD in IBD, and disease-specific modifiers may need to be added to present prediction models.

#### Markers of subclinical ASCVD

**Figure 2.**
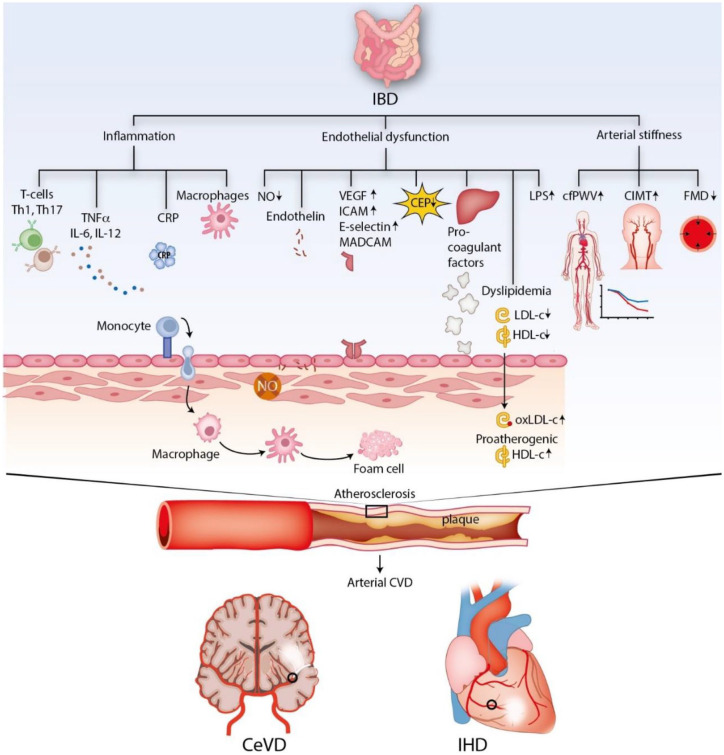
Immunologic profile and surrogate markers of subclinical atherosclerosis in IBD and the development of atherosclerosis. CEP, circulating endothelial progenitor cells; CeVD, cerebrovascular disease; cfPWV, carotid-femoral pulse-wave velocity; CIMT, carotid intima media thickness; CRP, C-reactive protein; CVD, cardiovascular disease; FMD, flow-mediated dilation; HDL-c, high-density lipoprotein cholesterol; ICAM, intracellular adhesion molecule; IBD, inflammatory bowel disease; IHD, ischemic heart disease; IL, interleukin; LDL-c, low-density lipoprotein cholesterol; LPS, lipopolysaccharides; MADCAM, mucosal addressin cell adhesion molecule; NO, nitric oxide; ox, oxidized; Th, T-helper cell; TNFα, tumor necrosis factor alpha; VEGF, vascular endothelial growth factor.

Several non-invasive modalities can be used to detect subclinical atherosclerosis, such as measurement of arterial stiffness by pulse-wave velocity between carotids and femorales (cfPWV; Δdistance/Δtime), the carotid intima media thickness (CIMT; mm) and flow-mediated dilation of arteries (FMD; % change in arterial dilation after acute bloodflow) by ultrasound, and coronary artery calcium (CAC) score by coronary computed tomography scans. The prevalence of subclinical atherosclerosis is probably increased in IBD patients, according to available data. For instance, the mean CIMT and cfPWV are significantly increased in IBD patients as compared with healthy controls, suggesting increased arterial stiffness.^87^ Likewise, the mean FMD was significantly lower in IBD patients as compared with the general population. Moreover, average coronary flow reserve (capacity of coronary circulation to respond to physiological increase in oxygen demand) is lower in IBD patients as compared with controls, and associated with inflammatory parameters and atherogenic lipid indices.^88^ In contrast to previous surrogate markers of subclinical atherosclerosis, preliminary data showed that CD patients without ASCVD or traditional risk factors do not exhibit higher CAC scores as compared age- and sex-matched controls.^89^ A relationship between arterial stiffness and inflammatory parameters has not been studied in IBD yet, but was confirmed in patients with other chronic inflammatory diseases, and healthy individuals.^90,91^ The added value of measuring markers and diagnosing subclinical atherosclerosis in routine IBD care has not been established (Figure 2).

#### Genetic factors

GWASs have linked nucleotide-binding oligomerization domain-containing protein 2 (NOD2) to the development of both CD and ASCVD. The NOD2 gene encodes for an intracellular receptor of peptidoglycan, a component of the bacterial cell wall, and three polymorphisms (R702W, G908R and 1007fs) have been shown to affect the intestinal mucosal barrier. NOD2 recognizes not only exogen ligands (microbial products) but also endogen ligands (oxidized LDL-c).^92,93^ Interactions between NOD2 and bacterial components also take place in the cytoplasm of endothelial cells and cardiomyocytes, illustrated by its response to one of the most frequently detected bacteria in atherosclerotic plaques, *Chlamydia pneumonia*.^94^ In mouse models, NOD2 loss of function has been shown to promote vascular inflammation underlying atherosclerosis.^95^ In a White population, an association between NOD2 polymorphisms and the development of clinically and angiographically evident CHD has been demonstrated, independent of cardiovascular risk factors (e.g. Leu1007fsinsC was associated with an increased risk of CHD, whereas GLY908ARG polymorphism was protective).^96^ In this analysis, NOD2 was more active in atherosclerotic plaques of the coronary arteries, although the three genetic variants associated with CD were not found to be associated with early-onset CHD.^97^ Whether genetic variants, either in NOD2 or currently unidentified genes, contribute to the susceptibility to ASCVD in IBD patients, need to be investigated in future GWASs.

### Pathogenesis

#### Inflammation and immune dysregulation

The pathogenesis underlying the association between IBD and ASCVD has not been fully elucidated. Recent advances in fundamental science show that low-grade chronic inflammation plays a role in all stages of atherosclerosis, from initiation to progression and subsequently thrombotic complications.^98^ The majority of immune cell types interacting in IBD were identified in the pathogenesis of atherosclerotic lesions, including macrophages, T and B cells, diverse pro- and anti-inflammatory cytokines and chemokines. CRP was detected in atherosclerotic lesions after binding to modified forms of LDL-c.^99^ Interleukin 6 (IL-6) activated immune cells involved in plaque formation and rupture. Both CRP and IL-6 were regarded as independent risk factors for CVD.^100^ TNFα induced cytokine release *via* monocyte activation, smooth-muscle-cell proliferation, and promoted the interaction between the endothelium and circulating leukocytes by the upregulation of adhesion molecules such as VCAM-1 and ICAM-1.^101,102^ Moreover, TNFα enhanced progression towards advanced atherosclerotic lesions in APOE*3 Leiden transgenic mice.^103^ Overexpression of TNFα led to the reduction of endothelium-dependent relaxation *in vitro* and *in vivo*.^51^

#### Mediator of endothelial dysfunction

Chronic low-grade inflammation influences the physical and functional properties of arteries, in specific, the vascular endothelium. The result is endothelial dysfunction (ED), characterized by increased vascular smooth muscle tone, upregulation of cellular adhesion molecules, leukocyte diapedesis and procoagulant activity, and known as an independent predictor of new-onset ASCVD.^104^ In IBD, ED is related to both the degree and duration of inflammation. Moreover, reduced production of the vasodilator nitric oxide (NO)^105–108^ and increase in the vasoconstrictor endothelin are reported in IBD.^109,110^ Both human and animal models provide evidence for upregulation of circulating and local intestinal adhesion molecules in IBD, together with vascular endothelial growth factor (VEGF), ICAM-1, MADCAM-1, and E-selectin.^111–113^ The expression of these molecules is correlated to disease activity, shown by a fall in the concentration of ICAM-1 and VCAM-1 after treatment.^114^ The expression of VEGF correlated with advancement of atherosclerotic lesions.^115^ The concentration of circulating endothelial progenitor cells (CEP), essential for endothelial repair, were decreased in both CD and UC patients as compared with healthy controls.^116^ Increased concentration of lipopolysaccharides (LPS), a microbial product that induces increased expression of pro-inflammatory cytokines that contribute to endothelial damage and foam-cell formation, were measured in UC and CD patients and correlated with CRP levels^117,118^ (Figure 2).

### Effect of IBD drugs on ASCVD

Interventions reducing inflammatory burden might attenuate ASCVD risk, as shown by reduced risk of IHD in patients with previous intestinal surgery and on combination therapy (IRR 0.92; 95%CI 0.75–1.13 and IRR 0.58; 95%CI 0.22–1.56, respectively).^72^

#### Aminosalicylates

Among 5-ASA users, a significant lower risk of IHD was reported as compared with non-users, also after adjustment for corticosteroid use.^72^ *In vitro*, 5-ASA shares anti-inflammatory and antioxidant properties with aspirin.^119,120^

#### Corticosteroids

Despite the frequent prescription of corticosteroids, remarkably few publications address the occurrence of hypertension, hyperglycemia, and insulin resistance specifically for IBD populations. A pilot study showed that orally taken but not bolus administration of methylprednisolone increased BMI, body fat percent and TC levels after 12-week treatment.^121^ In CD patients aged >50 years, treatment with corticosteroids significantly increased the risk of hypertension, but not of hyperglycemia or congestive heart failure.^122^ Studies addressing the risk of corticosteroid-induced diabetes in IBD are lacking. In a large population-based cohort study, the increased risk of DM type 2 was not assigned to corticosteroid exposure.^79^ In contrast, the risk of HF was higher in patients using systemic corticosteroids as compared with non-users (adjusted HR 2.51, 95%CI 1.93–4.57).^67^ A dose-dependent increase in the hazard of all-cause ASCVD was observed in immune-mediated diseases treated with corticosteroids, including IBD (1.08, 95%CI 1.07–1.10 per 5 mg/day).^123^ Future research is needed to establish whether these effects differ between formulas, among which corticosteroids with a different mechanism of action (prednisone *versus* budesonide) or mode of administration (oral *versus* topical).

#### Immunomodulators: methotrexate and thiopurines

The effect of methotrexate on cardiovascular risk factors and ASCVD incidence in IBD are unknown. In other chronic inflammatory conditions, methotrexate was shown to have a beneficial effect on arterial stiffness and to reduce cardiovascular events.^124^ Exposure to thiopurines was not associated with the risk of acute arterial events in IBD.^125^

#### Biologicals: TNFα antagonists, vedolizumab, ustekinumab

In a nationwide cohort study, exposure to anti-TNFα agents was associated with a decrease of the risk of acute arterial events as compared with unexposed patients (HR 0.79; 95%CI 0.66–0.95).^125^ Basic research reports that therapy targeting TNFα affects several risk factors associated with ASCVD.

First, the lipid profile is changed. The pro-atherogenic profile is improved during anti-TNFα therapy in both UC and CD. However, after initiation of TNFα antagonists, IBD patients displayed significantly higher small, dense LDL-c content within LDL-c fractions.^126^ A discrepancy exists between studies assessing the effect on absolute lipid levels. After a 14-week TNFα antagonist regimen, CD patients presented with increased levels of TC, HDL-c, and apo-A1.^127^ Long-term treatment of normolipidemic IBD patients showed persistent significant increase in TC and LDL-c after 3 years of follow up, resulting in an increased atherogenic index.^128^ Other studies reached contradictory results, and showed no difference between pre- and post-treatment lipid profiles.^128–130^

Second, while an increase in weight has been reported for all biologicals, TNFα inhibition increases abdominal fat tissue measured by magnetic resonance imaging, independent of BMI evolution.^129,131^ Third, a favorable effect on insulin sensitivity is described with declining glucose and glycated hemoglobin concentrations, probably as a consequence of the reversal of the TNFα-induced disturbance of insulin-mediated glucose uptake.^129,130^ Finally, antagonization of TNFα improves endothelial function, arterial stiffness and fibrinolysis.^52^

In the vedolizumab registration trials, cardiovascular death was reported in 1/895 (0.11%) UC and 1/1115 (0.09%) CD patients. In neither the *post hoc* analysis nor the long-term extension trials, additional cases were reported.^53,54^ Similarly, no cardiovascular events were found in pivotal trials of ustekinumab. After 3-year follow up, 1/567 (0.18%) CD patients presented with a cardiovascular event.^53^ Real-life data on cardiovascular risk in IBD patients treated with vedolizumab and ustekinumab are lacking, as well as fundamental research into the effect of these newer biologicals on processes associated with ASCVD pathogenesis.

#### Janus kinase inhibitors: tofacitinib

According to the registration trial data and available real-world cohort studies, major cardiovascular events during tofacitinib treatment occurred infrequently [incidence rate (IR) 0.24 per 100 person-years; 95%CI 0.07–0.62], and are not dose related.^132^ Four patients experienced major cardiovascular events (hemorrhagic stroke, acute coronary syndrome, MI and aortic dissection), of whom three out of four patients had several traditional cardiovascular risk factors. The incidence of major cardiovascular events was similar to that reported for other IBD drug therapies, and for tofacitinib in other chronic inflammatory conditions.^133,134^ Recent new safety data from the post-marketing trials in rheumatoid arthritis patients with high CV risk showed higher rates of major adverse cardiovascular events, for tofacitinib as compared with TNFα antagonists (IR 0.98, 95%CI 0.79–1.19 *versus* 0.73; 95%CI 0.52–1.01, respectively).^135^ Extrapolation of these data to UC patients seems premature, and long-term safety data are required.

A significant increase in TC, HDL-c, and LDL-c levels were observed during tofacitinib induction therapy in the phase III registration trials, which stabilized during maintenance therapy.^132^ These findings were confirmed in both retrospective and prospective real-life cohort studies.^136,137^ The lipid increases were modestly and reversibly correlated with CRP levels.^132^

### Effect of ASCVD drugs on IBD

#### Statins

Statins are among the most commonly prescribed agents worldwide. In addition to their lipid-lowering function, statins possess pleiotropic effects, including modulation of the immune system *via* inhibition of T cells, antigen presentation, and leukocyte infiltration in organs.^138^ A beneficial effect of statins on the risk of IBD and IBD flares is controversial, since conflicting data have been published.^139–143^ To date, these study results have no clinical consequence and statins are not used as drug therapy in IBD.

#### Antihypertensive drugs

Observational studies have reported a reduced risk of prescription of corticosteroids, hospital admission and intestinal surgeries in IBD patients using angiotensin-converting enzyme inhibitors (ACE-Is) and angiotensin II receptor blockers (ARBs).^144–146^ These findings need further confirmation. Inhibition of ACE and angiotensin receptors was shown to have anti-inflammatory effects. Possible mechanisms explaining the beneficial effect of ACE-Is and ARBs include the reduction of transforming-growth-factor-beta expression, micro- and macroscopic inflammation and intestinal fibrosis.^147,148^ In a histopathological study, higher concentrations of angiotensin I and II were measured in colonic biopsies of CD patients as compared with biopsies of UC patients and healthy controls, which correlated with the degree of macroscopic inflammation in CD (*r* = 0.86 and *r* = 0.68, *p* < 0.001, respectively). The mucosal levels of angiotensin I also correlated with the clinical Crohn’s Disease Activity Index.^149^ Several clinical studies report lower serum ACE levels in IBD patients as compared with healthy controls, irrespective of the presence of ACE polymorphisms, and mainly in patients suffering from ileitis.^150–152^ The ACE levels significantly raised in active CD patients achieving clinical remission.^150^ In line with this observation, decreased levels of ACE are hypothesized to be a result of local damage due to inflammation. Altogether, these results complicate the formulation of a unifying hypothesis behind these observations, which is potentially binomial with separate local and systemic effects.

### Guideline recommendations on ASCVD management

With regard to ASCVD, the ECCO guidelines state no specific recommendations except for management of known cardiovascular risk factors, including cigarette smoking cessation.^64^

Both in the American College of Cardiology/American Heart Association (ACC/AHA) and ESC guidelines, chronic inflammatory diseases are classified as risk factors for ASCVD.^65,153^ In these leading guidelines, IBD is not mentioned as an independent risk factor. The standard risk calculators for CVD risk prediction, such as the FRS and the ACC/AHA ASCVD risk calculator, are not validated for patients with chronic inflammatory diseases, and therefore may underestimate the risk for ASCVD. In the latest ESC guidelines, the recommendation is to use a 1.5 factor multiplier in assessing the 10-year CVD risk in rheumatoid arthritis patients.^154^ A gap in evidence is stated for other chronic inflammatory disease, including IBD. The ACC/AHA advice is to consider chronic inflammatory diseases as a risk-enhancing factor to guide decisions about preventive interventions (e.g. statin and antihypertensive therapy) in adult at borderline (5–7.5%) or intermediate (7.5–20%) 10-year ASCVD risk.

## Future research perspectives

Cardiovascular management in IBD requires a transition from reactive to proactive (preventive) care in high-risk patients. The efforts, costs, and burden for patients need to be well balanced against the benefit of preventive CVD measures for individual patients. Therefore, important knowledge gaps need further elucidation.

First, the complex, molecular pathophysiology of CVD in IBD needs further exploration, including immunological mechanisms involved in both IBD pathogenesis and atherogenesis. For instance, growing evidence suggests a causal link between host–gut microbiome interactions in both IBD and CVD risk factors, such as type 2 diabetes and metabolic syndrome.^155^

Second, clinical research needs to clarify the cardiovascular risk profile in IBD. Deep profiling of lipid changes and insulin resistance will add to better characterization of well-established CVD risk factors in IBD. Genetic studies should focus on variations associated with the development of CVD in IBD such as NOD2 gene polymorphisms. Patient registries and multidisciplinary collaborations enable the evaluation of epidemiology, clinical presentation and outcome of CVD in IBD, in comparison with the general population and other chronic inflammatory conditions. These insights are essential for the development of IBD-specific CVD prediction models.

Third, preventive strategies are likely to be dual: optimizing management of inflammation and cardiovascular risk management (CVRM). Adequate CVRM implies regular assessment, treatment and monitoring of CVD risk factors. The effect of CVD screening and subsequent intervention in IBD is yet unknown. Subsequently, it needs to be determined whether CVD screening in IBD proves cost effective.

Finally, when CVD risk warrants screening in clinical practice, CVRM in IBD needs to be well organized. The responsibilities of medical professionals working in primary and secondary care should be clarified in national guidelines.

## Conclusion

IBD is associated with a 2.5-fold increased risk of VTE, 1.2-fold increased risk of ASCVD, and 2-fold increased risk of HF as compared with the general population. VTE events stem from a hypercoagulable state in IBD patients, and risk factors include older age, active disease, more extensive disease, pregnancy, hospitalization, IBD-related surgery and use of certain drugs. Inflammatory processes play a role in the stepwise development of atherosclerosis. The active and self-perpetuating immune response in IBD could therefore increase the CVD risk. The risk of ASCVD is incompletely explained by the prevalence of traditional cardiovascular risk factors, but potentially associated with disease activity, female sex and changes of the lipid profiles. Inflammatory processes play a role in both chronic inflammation in IBD and the stepwise development of atherosclerosis. An active and self-perpetuating immune response could therefore increase the CVD risk as observed in IBD. In patients with IBD, markers of subclinical atherosclerosis are more frequently present. The lipid metabolism in IBD patients is characterized by lower levels of TC and LDL-c during active disease as compared with disease remission. During active inflammation, both HDL-c and LDL-c show pro-atherogenic properties. Although the increased risk of CVD is recognized in IBD, preventive management may vary across clinical practices, especially for the situations for which specific recommendations are lacking in guidelines, such as ASCVD. To improve ASCVD prognosis in IBD, ongoing scientific research focuses on unraveling the pathogenesis and the cardiovascular risk profile, including both traditional and IBD-specific risk factors, underlying the increased risk of ASCVD in IBD.
